# CD 200 – A useful marker in chronic B lymphoprolipherative disorders

**Published:** 2012

**Authors:** A Iova, A Vlădăreanu, H Bumbea, M Begu, D Vasile, E Andruş

**Affiliations:** Department of Hematology, University Emergency Hospital, “Carol Davila” University of Medicine and Pharmacy, Bucharest

**Keywords:** CD200, chronic lymphocytic leukemia, mantle cell lymphoma, chronic lymphoproliferative disorders

## Abstract

**Background**: The diagnosis and management of the patients with chronic lymphoproliferative diseases have become dependent on immunological criteria. Flow cytometry immunophenotyping is used for rapid and specific diagnosis but there are cases when we are not facing a typical immunophenotype, so there is a constant need to find new markers and new combinations of markers that would allow the improvement and the development of our diagnosis.

**Aim:** Our aim was to evaluate CD 200 expression in different B-cell chronic lymphoproliferative disorders. CD200 is a membrane glycoprotein belonging to the immunoglobulin superfamily and the over-expression of CD200 has been reported in a number of malignancies, including CLL, as well as on cancer stem cells.

**Methods:** We analyzed the CD200 expression in 122 patients diagnosed with chronic lymphoproliferative disorders (100 patients with CLL, 10 patients with splenic marginal zone lymphoma (SMZL), 10 patients with MCL and 2 patients with hairy cell leukemia), in the Department of Hematology of the University Emergency Hospital, Bucharest. We performed immunophenotypical analysis of peripheral blood and bone marrow aspiration on BD FACS Calibur flowcytometer.

**Results:** CD200 was brightly expressed in all 100 CLL patients (100%). In SMZL patients, CD200 was dim positive (40%-60%), in patients with HCL. CD200 was also bright positive (96% and 97%) and in patients with MCL CD200 was negative (1-10%); CD 200 was significantly higher in CLL patients compared with other B-cell chronic lymphoproliferative disorders. We found 14 patients with CD19, CD5 positive population and CD23- , but with high expression of CD 200. Cyclin D1 was negative on bone marrow biopsy in 13/14 of these patients. (1/14 patients were without bone marrow involvement);

**Conclusions:** CD200 has a great impact in diagnosing B- chronic lymphoproliferative disorders, especially when we want to determine the origin of a CD19, CD5 positive population and distinguish between CLL and MCL. CD 23 is a reliable marker in those cases, but, as we showed, CD23 might have a lower specificity than CD200 for CLL. We added CD200 in our panels in order to diagnose chronic lymphoproliferative disorders, not to replace CD 23, but to improve and save time in our diagnostic process. The high expression of CD200 in CLL and HCL could open the option for new- targeted therapy (anti-CD200).

Chronic B-cell lymphoproliferative disorders (B-CLPD) are a biologically heterogeneous group of malignant diseases characterized by an accumulation of mature B-lymphocytes in the bone marrow (BM), peripheral blood, and lymphoid tissues [**1**]. B-CLPD is now most often diagnosed by flow cytometric immunophenotyping that identifies a clonal light-chain restricted population expressing B-cell markers in the blood or BM [**2**]. Flow cytometry immunophenotyping is used for rapid and specific diagnoses. We used a panel of surface markers that gives us information about the lineage, percentage or aberrant expression of the population under discussion, in screening, lymphocytosis and in diagnosing malignant lymphoproliferation by flow cytometry.

Flow cytometric identification of physiological as well as malignant cell populations of lymphocytes, mainly, depends on the recognition of specific antigen patterns in combination with light scatter signals [**3**]. Sensitivity and specificity of the method increase along with the information that could be obtained simultaneously on the single cell level [**4,5**].

Therefore, there is a constant need for finding new markers and new combinations of markers that allow us to improve and develop our diagnoses.

Patients with B-CLPDs can be further subclassified according to the expression of CD5 [**6**]. Those with a B-CLPD that does not express CD5 will usually be found to have the leukemic phase of a well-defined lymphoma such as marginal zone lymphoma (MZL), lymphoplasmacytic lymphoma (LPL), follicular cell lymphoma (FCL), or hairy cell leukemia (HCL) [**7**]. B-CLPD patients who express CD5 are usually patients with chronic lymphocytic leukemia (CLL) or mantle cell lymphoma (MCL), which is rarely met the marginal zone lymphoma. CD5 expression in B-CLPD is not specific for CLL or MCL and has been reported to occur in 5 to 10% of LPL and 20% or more of MZL [**4,8,9**].

The main immunophenotypic features that define B cell chronic lymphoproliferative diseases (Cheson) are summarized in **Table 1**.

**Table 1.** The main immunophenotypic features

**Marker**	**CLL**	**PLL**	**HCL**	**HCL-V**	**SLVL**	**FL**	**MCL**
**CD19**	++	++	+++	+++	++	++	++
**CD20**	Dim	+++	+++	+++	++	++	++
**sIg**	Dim	+++	+++	+++	++	++	++
**CD5**	++	-/+	-	-	-/+	-	++
**CD10**	-	-/+	-	-	-/+	++	-
**CD11c**	-/+Dim	-/+	++	++	+/-	-	-
**CD22**	-/+Dim	++	+++	+++	++	++	++
**CD23**	++	-/+	-	-	-/+	-	-
**CD25**	-/+	+/-	+++	-	+/-	-	-
**CD38**	-/+	+/-	-/+	-	-/+	-/+	-
**CD79a**	-	++	++	++	++	++	++
**CD103**	-	-	+++	+++	+/-	-	-

One of the major immunophenotypic differences between CLL and MCL is the CD23 status. Before the anti-cyclin D1 antibody was widely available as a powerful tool to help distinguish MCL from CLL by flow cytometry, CD23 status had been considered very useful in separating CLL from MCL, especially when CD23 is either moderately. to brightly positive or completely negative by flow. cytometry [**10**]. CD200 is a new marker that was found to be up-regulated in chronic lymphocytic leukemia (CLL)/small lymphocytic lymphoma (SLL) when compared with normal B cells by flow cytometry immunophenotyping [**11**].

CD200 (OX-2 antigen) is a type I immunoglobulin superfamily membrane glycoprotein 1, widely expressed in multiple cell types, including B cells, a subset of T cells, dendritic cells, endothelial cells, and are also located in the peripheral and central nervous system [**12**]. CD200 interacts with CD200R, an immunoglobulin superfamily inhibitory receptor, expressed primarily on myeloid/monocyte lineage cells, and has a suppressive effect on T cell–mediated immune response [**13,14**]. CD200 expression by neoplastic cells down-regulates the TH1 immune response and suppresses the antitumor immune response in an animal model of CLL [**15,16**].

## Material and Methods

We analyzed CD200 expression in 122 patients diagnosed with chronic lymphoproliferative disorders (100 patients with CLL, 10 patients with splenic marginal zone lymphoma (SMZL), 10 patients with MCL and 2 patients with hairy cell leukemia), in the Department of Hematology of the University Emergency Hospital, Bucharest. Tests included only patients, without any previous therapy for the B lymphoproliferative disorder. We performed an immunophenotypical analysis of peripheral blood and bone marrow aspirate on BD FACS Calibur flowcytometer. According to EGIL/WHO recommendations, our diagnose panel included the following markers: CD19, CD20, CD5, CD23, CD79B, CD103, CD11c, CD25, CD10, FMC7, CD38, IgM, IgG, IgD, KAPPA/LAMBDA chains. The diagnosis was completed by hystological and immunochemical analysis.

## Results

We included patients aged 45 to 81 years, with a male-to-female ratio of 1,65/1;

**Fig. 1:**
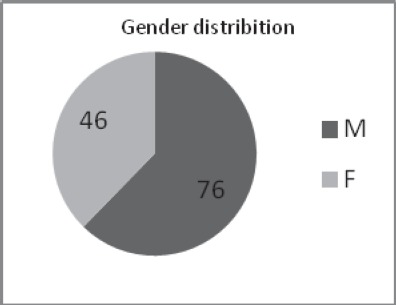
Gender distribution

**Fig. 2:**
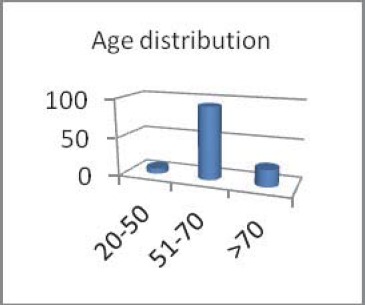
Age distribution

**Fig. 3:**
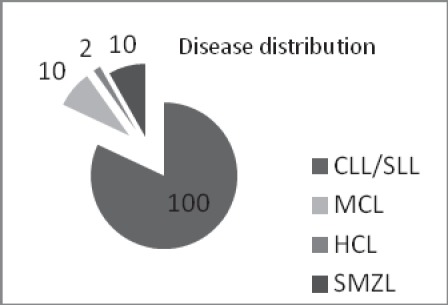
Disease distribution

Hemathological parameters:

**Table:** 

WBC count* 10^3^/μL(range)	18,4(1,9-358)
Hb level g/dl (range)	12,3(5,6-15,1)
Platelet count* 10^3^/μL (range)	158(23.000-571)
Lymphocytosis %	42%
AIHA	6,3%
**BM infiltration %	52%(3-98)

Data showed as median (minim, maxim)

**Assessed by bone marrow aspirate or biopsy or both

We performed a 4-color analysis on a Facs Calibur Flowcytometer as follows:

-Paint a gait/Cellquest gating strategy:

• First gating=CD 45
• One B cell marker in every tube (CD19/CD20/CD22)=>isolation of B cell total population =4 color strategy limitation

**Fig. 4:**
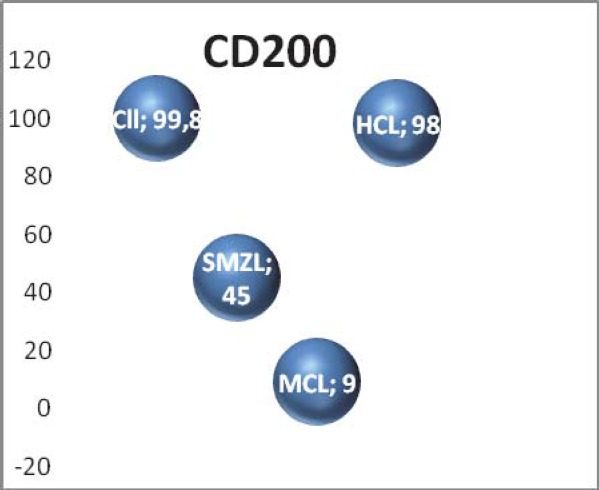
CD 200 expression in our groups of patients

CD200 was brightly expressed in all 100 CLL patients (100%). In SMZL patients, CD200 was dim positive (40%-60%), in patients with HCL,CD200 was also bright positive (96% and 97%) and in patients with MCL CD200 was negative (1-10%); CD200 was significantly higher in CLL patients compared with the other B-cell chronic lymphoproliferative disorders (**Fig. 4**).

**Fig. 5:**
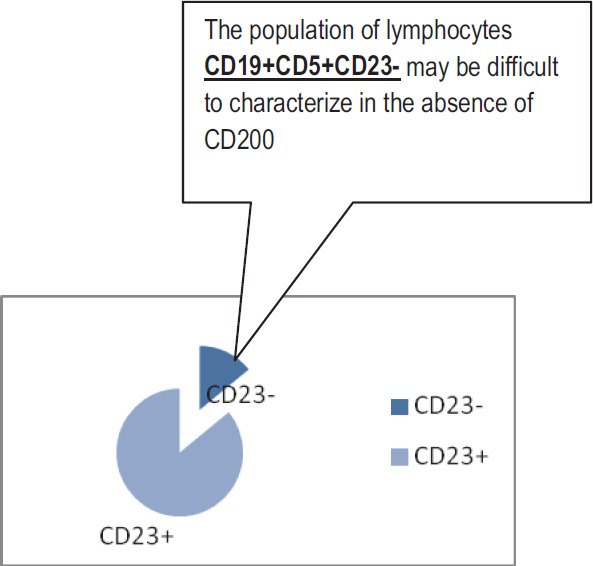
The expression of CD23 in the Cll/Sll group;

We found 14 patients, who represented 14% from the CLL/SLL group, with CD19, CD5 positive population and CD23 (**Fig. 5**), but with high expression of CD 200. Cyclin D1 was negative on bone marrow biopsy in 13/14 of these patients (1/14 patients was without bone marrow involvement);

**Fig 6.**
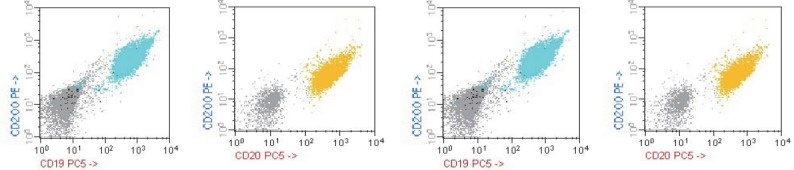
FACS – Calibur acquisiyion, CellQuest software

The expression of CD200 in hematologic malignancies was first reported for chronic lymphocytic leukemia (CLL) [**17**]. We have studied the expression of CD200 in various lymphoid malignancies.

## Discussion

We have studied the expression of CD200 in various lymphoid malignancies. In this report, we confirm some of literature data regarding CD200, CD200 is uniformly expressed in CLL, whereas its expression is not detected in MCL.

The expression of CD200 in hematologic malignancies was first reported for chronic lymphocytic leukemia (CLL) [**17**]. CD200 is very helpful when we want to characterize a clonal population CD19+CD5-CD23-. As we have showed, CD200 is negative in MCL, positive low in SMZL and high positive in HCL and CLL.

## Conclusions

CD200 has a great impact in diagnosing B-chronic lymphoproliferative disorders, especially when we want to determine the origin of a CD19, CD5 positive population and differentiate CLL from MCL. CD23 is a reliable marker in the presented cases, but, as we showed, CD23 might have a lower specificity than CD200 for CLL. Also, the other markers (CD20, CD79b, with a higher expression in NHL then in CLL/SLL), play an important role in the diagnosis but they are not as specific markers as CD23. The diagnosis of MCL is confirmed with the help of cyclin D1 positivity, by the presence of the t(11;14)(q13;q32) chromosomal translocation detected by cytogenetics by Western blot or by Polymerase Chain Reaction (PCR) analysis or by fluorescence in situ hybridization (FISH). Still, these methods are expensive, time-consuming and not always available.

We added CD200 in our panels for diagnoses of chronic lymphoproliferative disorders, with the purpose not to replace CD23, but to improve and save time in our diagnoses. The high expression of CD200 in CLL and HCL can give new directions for new-targeted therapy (anti-CD200).
